# A Case of Fastidious Mycobacterium chelonae-Causative Cellulitis: Clinical Manifestations of a Rare Bacterial Infection

**DOI:** 10.7759/cureus.25816

**Published:** 2022-06-10

**Authors:** Grant E Gregory, Hannah M Gregory, Mo Zaman, William D Hewitt

**Affiliations:** 1 Medicine, Alabama College of Osteopathic Medicine, Dothan, USA; 2 Anesthesiology and Critical Care, Alabama College of Osteopathic Medicine, Dothan, USA; 3 Internal Medicine, Southeast Health Medical Center, Dothan, USA; 4 Infectious Disease, Southeast Health Medical Center, Dothan, USA

**Keywords:** skin disease/ dermatology, infectious disease pathology, acid fast bacilli (afb), cellulitis, non-tuberculoid mycobacterium

## Abstract

Cellulitis is rarely caused by the organism *Mycobacterium chelonae* (*M. chelonae*). In this case report, we detail the clinical course of a 43-year-old female with persistent cellulitis in her right lower extremity despite intensive empiric therapy. The patient was formally diagnosed with *Mycobacterium chelonae*-causative cellulitis after an extensive workup including a prolonged hospital stay involving surgical biopsy and a delayed result of an acid-fast bacilli stain. The patient was treated appropriately, including a complete resolution of symptoms, with an extended regimen of clarithromycin and doxycycline to target *M. chelonae*.

## Introduction

Cellulitis, a localized infection of the deep dermis and subcutaneous tissue, is a common medical diagnosis resulting in thousands of hospital admissions and billions of dollars in treatment costs annually [1]. Non-purulent cellulitis is most commonly caused by *beta-hemolytic Streptococcus*, including *Group A Streptococcus* (GAS) and *Group B Streptococcus* (GBS), and occasionally *Group C* and *Group F Streptococcus* [2]. Clinical symptoms of cellulitis include erythema, edema, warmth, and tenderness of the extremity or affected body region [3]. These are often associated with the systemic findings of fevers and chills with occasional nausea and vomiting. While regarded as a clinical diagnosis, cellulitis is prone to misdiagnoses, occurring in approximately 31% of cases according to one case series [4]. Thereby, the true prevalence of cellulitis may be misrepresented. Cellulitis is generally treated empirically with antibiotics covering GAS and methicillin-sensitive* Staphylococcus aureus* (MSSA), while occasionally warranting additional coverage of methicillin-resistant* Staphylococcus aureus* (MRSA) in at-risk populations [5]. Some cases of persistent cellulitis, despite antibiotic therapy, may be attributed to rare organisms [5].

*Mycobacterium chelonae *(*M. chelonae*) is one of the rare organisms that can cause cellulitis [2]. *M. chelonae*, a subspecies of nontuberculous mycobacterium (NTM), is rarely identified in 0.08 to 0.2 cases per 100,000 people with cellulitis [2]. In this case report, we detail the clinical course and prolonged hospital stay of a 43-year-old female with persistent cellulitis, despite intensive empiric therapy. The causative organism was eventually identified by culture as *M. chelonae* and treated successfully with appropriate antibiotic therapy.

## Case presentation

A 43-year-old immunocompetent female with a past medical history of congestive heart failure (CHF) and chronic kidney disease (CKD) presented to the emergency room (ER) with a cough and shortness of breath. She also complained of right-lower extremity (RLE) swelling, redness, and pain.

**Figure 1:**
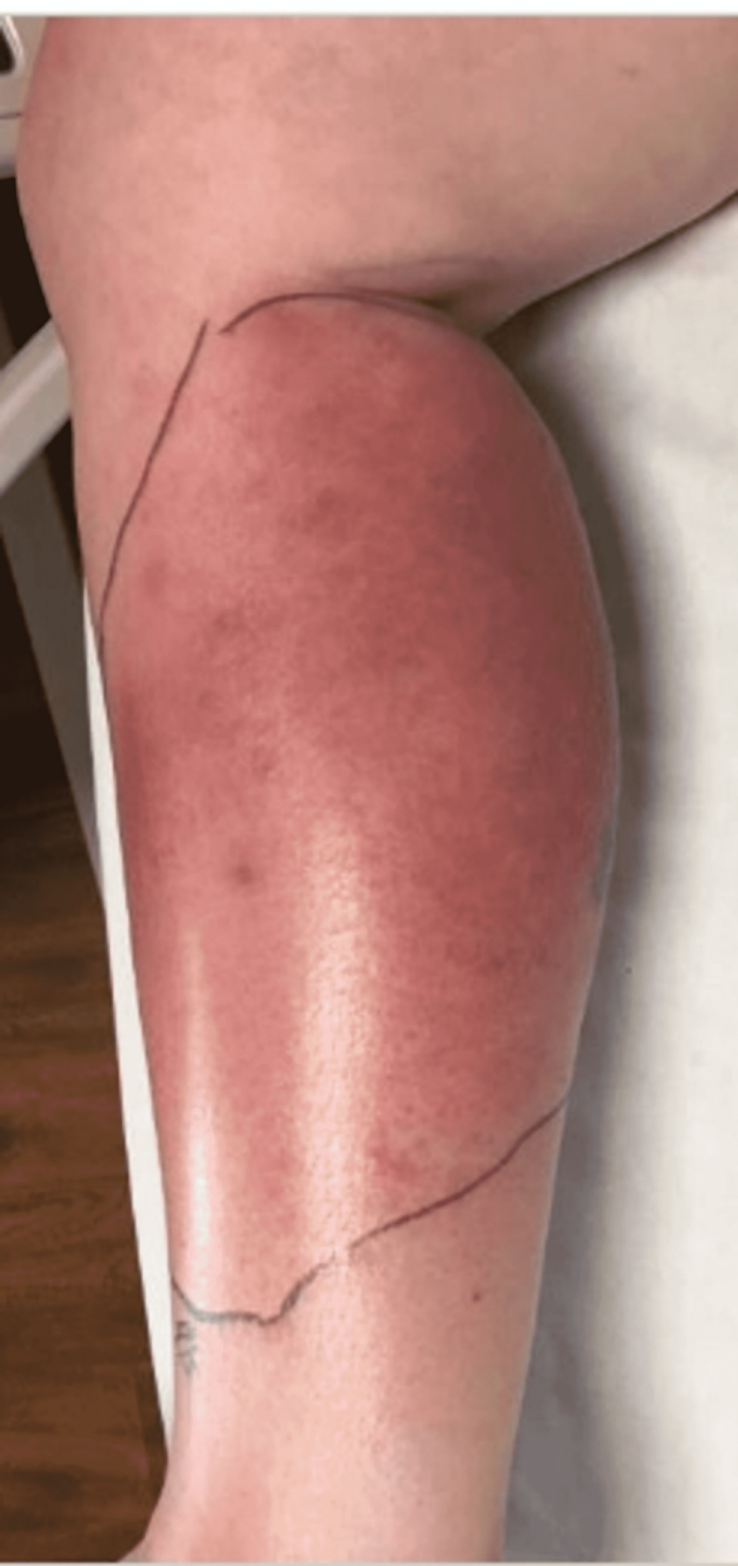
Photo demonstrating the patient’s cellulitis of her RLE during her first hospital stay. RLE: Right-lower extremity

**Figure 2:**
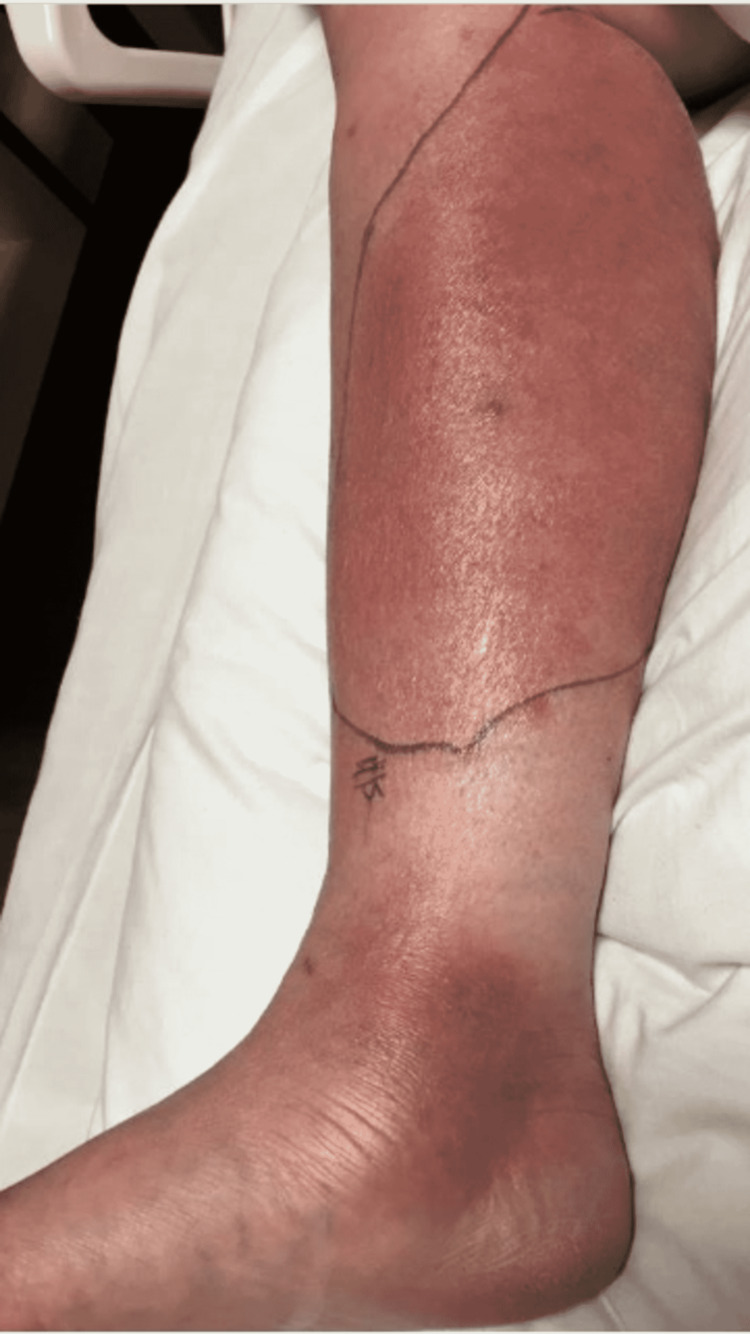
Photo demonstrating the patient’s cellulitis of her RLE during her first hospital stay. RLE: Right-lower extremity

Upon emergency room arrival, the patient was in obvious acute distress from her pain. Her vitals were as followed: heart rate (HR) of 91, respiratory rate (RR) of 23, blood pressure (BP) 142/84, and temperature of 97.4 Fahrenheit. She was tachypneic but not using accessory muscles to breathe. On lung auscultation, she had rales at the lung bases, but no wheezing or rhonchi was appreciated. Cardiac auscultation revealed normal heart sounds with a regular rate and rhythm without any murmurs, rubs, or gallops. An abdominal exam was benign. Her RLE had patchy erythema with skip lesions extending proximally from the level of the malleolus to just beneath her patella and encircling the entire area, as demonstrated in Figure 1 and Figure 2. This was also extremely tender to even the lightest of touch. There was no fluctuance felt on palpation.

Laboratory results revealed evidence of leukocytosis with a white cell count of 12,400 and lactic acidosis. Given her respiratory rate and her leukocyte count, the patient met the criteria for systemic inflammatory response syndrome (SIRS) from suspected cellulitis, and she was admitted to the medical floor for a sepsis workup.

Two sets of blood cultures were obtained, and the patient was then started on empiric antibiotics with intravenous (IV) clindamycin 600 mg every 8 hours and IV ceftriaxone 1 g every 24 hours. An electrocardiogram revealed a normal sinus rhythm. Venous ultrasound of the RLE ruled out any evidence of deep vein thrombosis, and non-vascular ultrasound of the RLE was unremarkable for soft tissue swelling or abscess formation. Finalized blood cultures remained negative, and the antibiotic regimen was continued empirically to treat the persistent cellulitis.

After five days of the antibiotic regimen with clindamycin and ceftriaxone, the patient’s cellulitis was worsening rather than improving in both symptoms and appearance. An infectious disease consult was obtained, and the decision was made to discontinue clindamycin and initiate IV vancomycin at 750 mg every 12 hours. After five days of the combined ceftriaxone and vancomycin regimen (10 total days of antibiotic therapy), the patient’s cellulitis showed no improvement despite an improvement in her white blood cell (WBC) count. A magnetic resonance image (MRI) was subsequently performed which ultimately ruled out myositis, osteomyelitis, and abscess formation.

**Figure 3:**
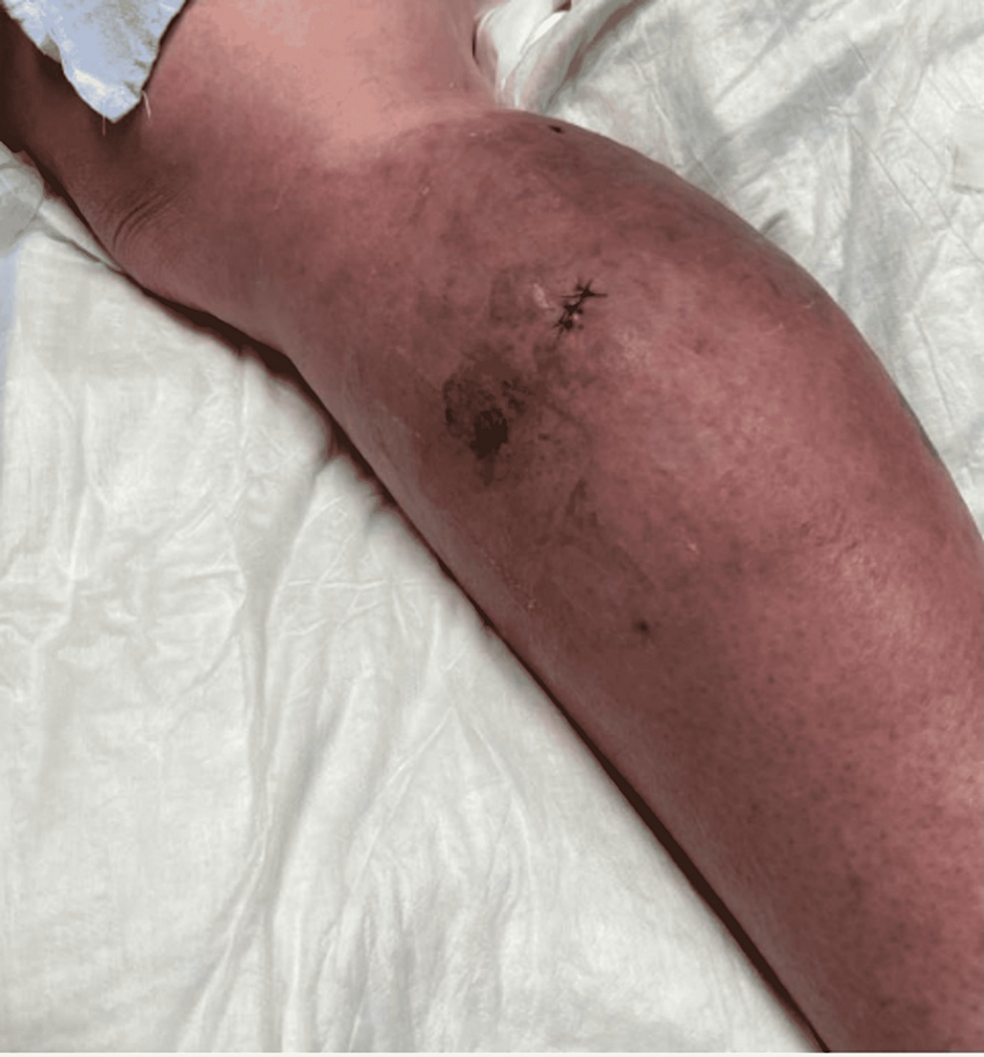
Post-surgical RLE photo demonstrating mild improvement of the cellulitis compared to admission. The decision was made to return home in the following days. RLE: Right-lower extremity

On day 10 of the patient’s hospital stay, the ceftriaxone and vancomycin regimen was completed without clinical improvement. IV piperacillin/tazobactam was then started at 4.5 g every 8 hours for broadened coverage. General surgery was consulted to obtain a skin biopsy for bacterial, fungal, and AFB cultures and pathology. The tissue sample was sent to a specialty lab for an acid-fast bacilli (AFB) stain and culture as well. In-house results of the samples taken were negative for AFB stain, fungal elements, and bacterial culture. Histopathology of the RLE biopsy was evaluated as “acute-on-chronic dermatitis.” Piperacillin/tazobactam was discontinued after seven days due to the development of a moderately-severe drug rash. The drug rash resolved after discontinuation, and her cellulitis remained clinically stable in both symptoms and appearance as seen in Figure 3. After a 17-day hospital course with no worsening of the patient’s cellulitis, she made the decision to be discharged with a final diagnosis of acute on chronic dermatitis.

**Figure 4:**
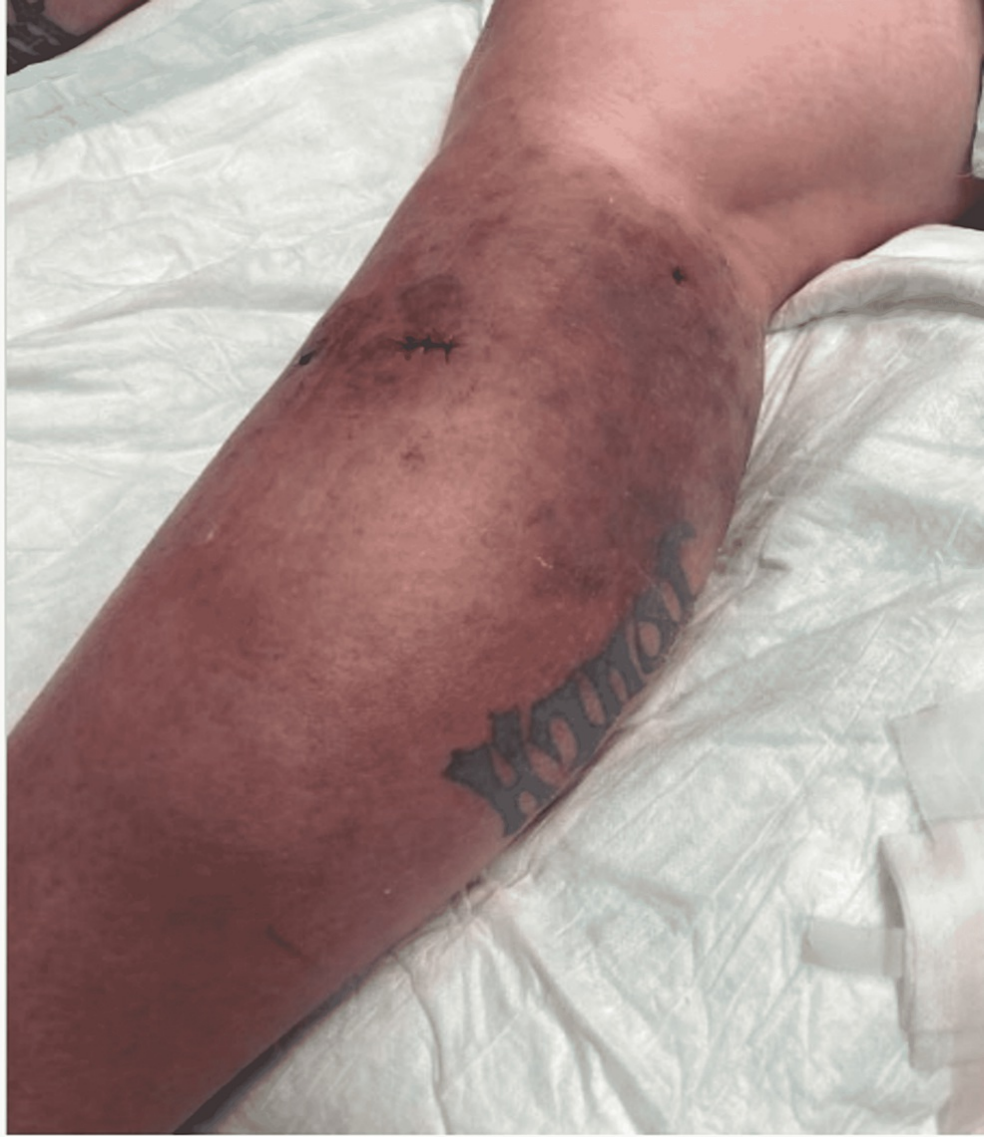
Photo of the RLE showing cellulitis upon admission for the patient’s second hospital stay. RLE: Right-lower extremity

**Figure 5:**
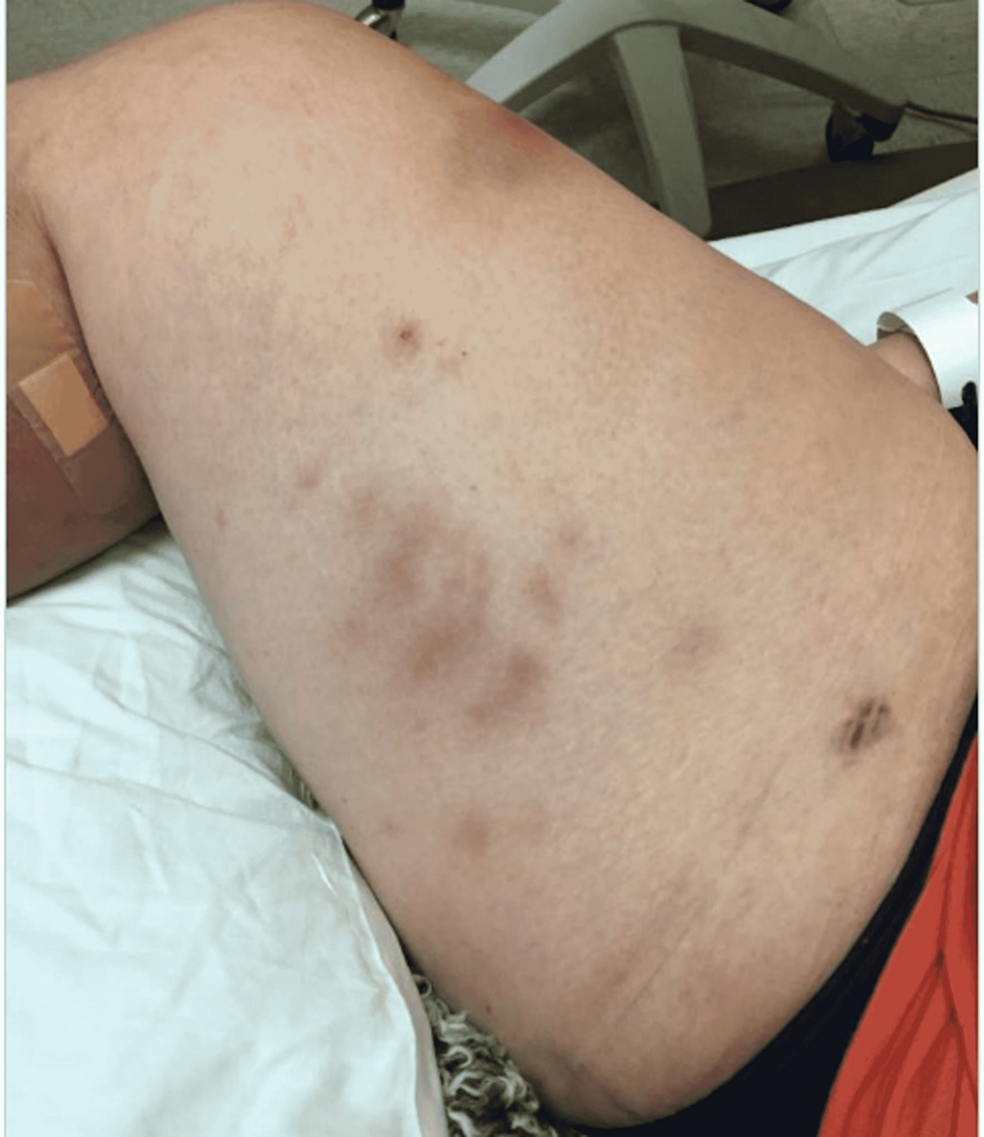
Photo of the RLE showing cellulitis upon admission for the patient’s second hospital stay. Note the proximal progression of the lesions. RLE: Right-lower extremity

With progressively worsening and unbearable pain in her RLE, the patient returned to the ER one day after her initial discharge. She was admitted for worsening cellulitis symptoms. The cellulitis had not improved and lesions were progressing proximally up the thigh, as evidenced in Figure 4 and Figure 5. An infectious disease team was again consulted during this second admission. Still awaiting a formal report from the state lab for AFB stain and culture results, a phone call was made, and it was confirmed that the AFB stain was indeed positive. With this new information, the patient was empirically started on clarithromycin 500 mg every 12 hours and doxycycline 100 mg every 12 hours to cover for atypical Mycobacteria. A QuantiFERON gold test was performed to rule out Mycobacterium tuberculosis, which was negative. After just a few days of treatment with clarithromycin and doxycycline, the patient’s cellulitis was clinically improving. She reported improvement in her RLE pain for the first time since the process had started. At the time of her second discharge, the specialty state lab formally reported positivity for an AFB stain. She was sent home on a 3-6-month course of oral antibiotic therapy of clarithromycin and doxycycline. Approximately one month after the initial skin biopsy, a culture report confirmed the growth of *Mycobacterium chelonae*, which is an atypical mycobacterium and rare causative organism of cellulitis.

**Figure 6:**
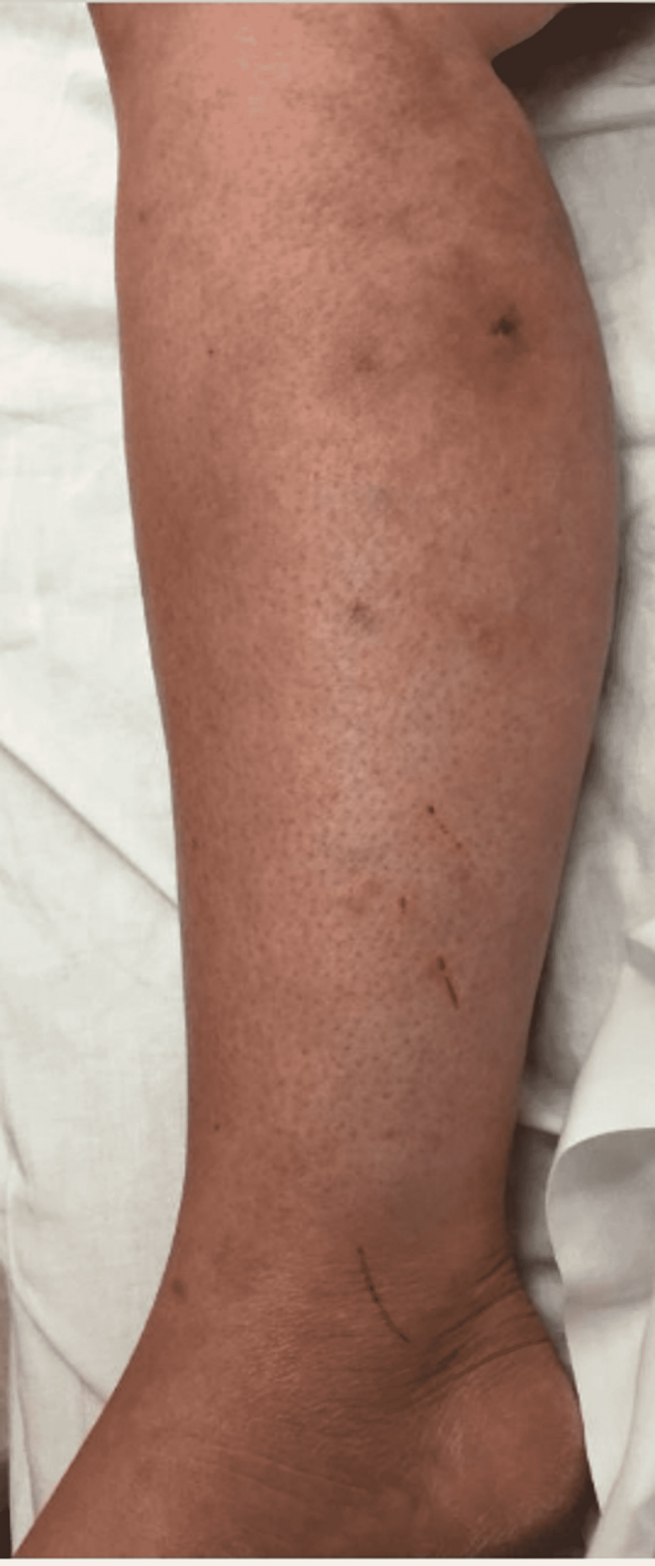
Photo demonstrating clinical improvement of the patient's RLE cellulitis on her second hospital discharge. RLE: Right-lower extremity

**Figure 7:**
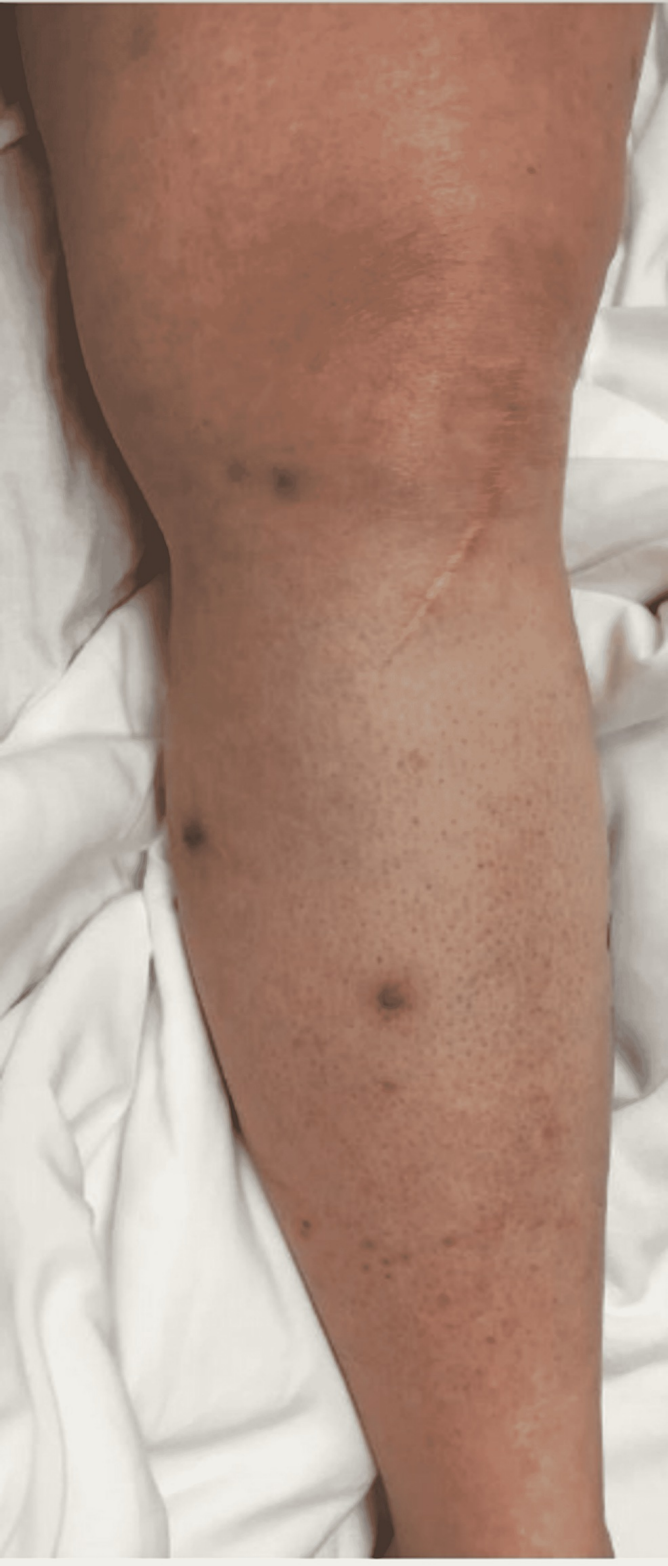
Near-complete resolution of the cellulitis on follow-up five weeks after her third and final discharge.

One month following her second hospital discharge, the patient returned to the emergency room for the third time with complaints of pain, purulent drainage from her biopsy site, and erythema of her RLE despite compliance with her antibiotic regimen, as photographed in Figure 6. She was informed that successful treatment usually requires months of prolonged therapy with antibiotics that cover NTMs. The clarithromycin and doxycycline regimen was continued and the patient was discharged from the emergency room. The patient did not return to the hospital with further complaints of her cellulitis in the following months. At a five-week follow-up appointment with primary care, the patient's cellulitis had nearly resolved, as demonstrated in Figure 7. The decision was made to continue antibiotic therapy with the aim of completely eradicating the rare mycobacterium.

## Discussion

Mycobacterial etiologies are subdivided into three species: *Mycobacterium tuberculosis complex* (*M. tuberculosis, M. bovis, M. leprae*), *Mycobacterium avium complex* (MAC), and non-tuberculous mycobacteria (NTM). *M. chelonae* represents one of at least 150 distinct species of NTM [6]. Human-to-human transmission does not occur with NTMs, unlike other species of mycobacterial complexes. Rather, NTMs exist freely in the environment and are transmitted through environmental contact [6]. Rates of NTM infection appear to be higher in the southeastern United States, perhaps due to an environment most suitable for NTM viability [7]. NTMs can be diagnosed with an AFB stain after ruling out tuberculoid and avium complexes. Specific NTM identification is revealed with bacterial culture. Historically classified as either rapid-growing, medium-growing, or slow-growing, *M. chelonae* represents a rapidly-growing subspecies of NTM. Today, laboratories are increasingly utilizing molecular testing to rapidly identify NTM subspecies [6,8].

*M. chelonae*, a gram-positive rod, most commonly affects the skin and should be considered as a potential causative organism in patients with long-term intractable skin lesions whose symptoms do not improve with standard empiric antibiotic therapy. *M. chelonae* most commonly affects the extremities due to preferential growth at lower temperatures [2]. *M. chelonae* are universally resistant to cefoxitin, but equally susceptible to tobramycin and clarithromycin [2]. In our case, the patient was effectively treated with clarithromycin after the identification of *M. chelonae*. Linezolid and imipenem have also shown moderate effectiveness in limited evaluation [2].

To study the longevity required to successfully treat *M. chelonae*, one case series noted an average resolution time of *M. chelonae* to be approximately 24 weeks while on clarithromycin [6]. The American Thoracic Society (ATS) recommends 4-6 months of treatment for NTM with serial monitoring of the patient’s clinical status to assess for improvement [9]. Patients should be counseled on the expected prolonged course of therapy and thus the importance of compliance that is necessary to successfully treat *M. chelonae*.

Differential diagnoses of intractable skin lesions with standard empiric treatment include actinomycosis, blastomycosis, histoplasmosis, nocardiosis, and mycetoma, in addition to species of nontuberculous mycobacteria [2]. Our case was complicated by an initial negative AFB stain, potentially indicating the need for improved training and distribution of resources for diagnosing rare bacteria. Local cultures did reveal *M. chelonae* weeks into an extended patient stay, equally highlighting the fastidious nature of *M. chelonae*. A skin biopsy may be the only reliable way to make a diagnosis, as there is often no active drainage. Specimens may need to be sent to specialty labs throughout the country for culture and sensitivities.

## Conclusions

Although exceedingly rare, *M. chelonae* is an etiologic agent of cellulitis in both immunocompetent and immunocompromised patients, particularly in patients engaging in activities with increased inoculation risk such as punctures. Consideration is warranted in patients with persistent cellulitis who have unchanged or worsening symptoms despite empiric antibiotic therapy. Treatment of suspected *M. chelonae* should be initiated with clarithromycin and confirmed with culture and sensitivities.
